# A functional food approach: probiotic-fermented *Angelica* spp. improves skin hyperpigmentation via gut-skin axis and its microbial metabolite hippuric acid

**DOI:** 10.3389/fnut.2026.1890406

**Published:** 2026-08-18

**Authors:** Yuyuan Li, Dandan Xiang, Xingda Huang, Jixiang Li, Xuxu Du, Donglin Yuan, Chenglong Hou, Qian Wang, Yuanhang Wu, Binbin Hou, Chaoran Wang, Yixin Ren, Ming Li

**Affiliations:** 1 Advanced Institute for Medical Sciences, Dalian Medical University, Dalian, China; 2 College of Basic Medical Science, Dalian Medical University, Dalian, China; 3 Department of Laboratory, Qingyang People’s Hospital, Qingyang, China; 4 CapitalBio Technology (Liaoning) Co., Ltd., Dalian, China; 5 Department of Oncology, The First Affiliated Hospital of Dalian Medical University, Dalian, China; 6 Department of Dermatology, The Second Affiliated Hospital of Dalian Medical University, Dalian, China; 7 CAS Key Laboratory of Separation Science for Analytical Chemistry, Dalian Institute of Chemical Physics, Chinese Academy of Sciences, Dalian, China

**Keywords:** *Angelica dahurica*, *Angelica sinensis*, gut-skin axis, hippuric acid, melanogenesis, probiotic fermentation

## Abstract

**Background:**

*Angelica dahurica* (Bai-zhi) and *A. sinensis* (Dang-gui) are traditional food-medicines used for skin disorders, but the mechanism of their oral efficacy remains unclear. Probiotic fermentation is a promising strategy to enhance the bioactivity of plant-based foods.

**Objective:**

This study aimed to investigate the functional nutritional properties of probiotic-fermented *Angelica* spp., evaluate its anti-melanogenic efficacy, and elucidate the role of gut microbiota remodeling and the microbial metabolite hippuric acid (HA) in mediating these effects via the gut-skin axis.

**Methods:**

The aqueous extract of *A. dahurica* and *A. sinensis* was fermented with *L. plantarum*. The fermented broth (FB) was chemically profiled and assessed for antioxidant and anti-melanogenic activity in B16F10 cells. Its efficacy was evaluated in a UVB/progesterone-induced hyperpigmentation mouse model treated with FB orally (GI) or topically (EI). Gut microbiota (16S rRNA sequencing) and metabolomic profiles were analyzed. The role of the key metabolite, hippuric acid (HA), was further investigated *in vitro* using HT-29 cells.

**Results:**

Fermentation enriched bioactive constituents such as salicylic acid and vitamin B2. FB exerted direct anti-melanogenic effects in vitro and exhibited notable in vitro antioxidant activity. *In vivo*, it relieved UVB/progesterone-induced hyperpigmentation primarily by remodeling gut microbiota and boosting circulating HA. HA modulates transcription of intestinal immune-related genes including NR1H4 and IL-1β, and its concentration positively correlated with beneficial gut taxa. This metabolite alleviates systemic inflammatory signals that promote melanin overproduction via regulating intestinal immune programs.

**Conclusion:**

Our findings reveal that probiotic fermentation transforms traditional food-grade *Angelica* spp. into a potent functional food. Its skin-beneficial effect is mediated by the gut microbiota-derived metabolite HA, highlighting a novel mechanism by which dietary phytochemicals can improve skin health via the gut-skin axis and offering a scientific basis for the modern application of traditional foods.

## Graphical abstract

**Figure:**
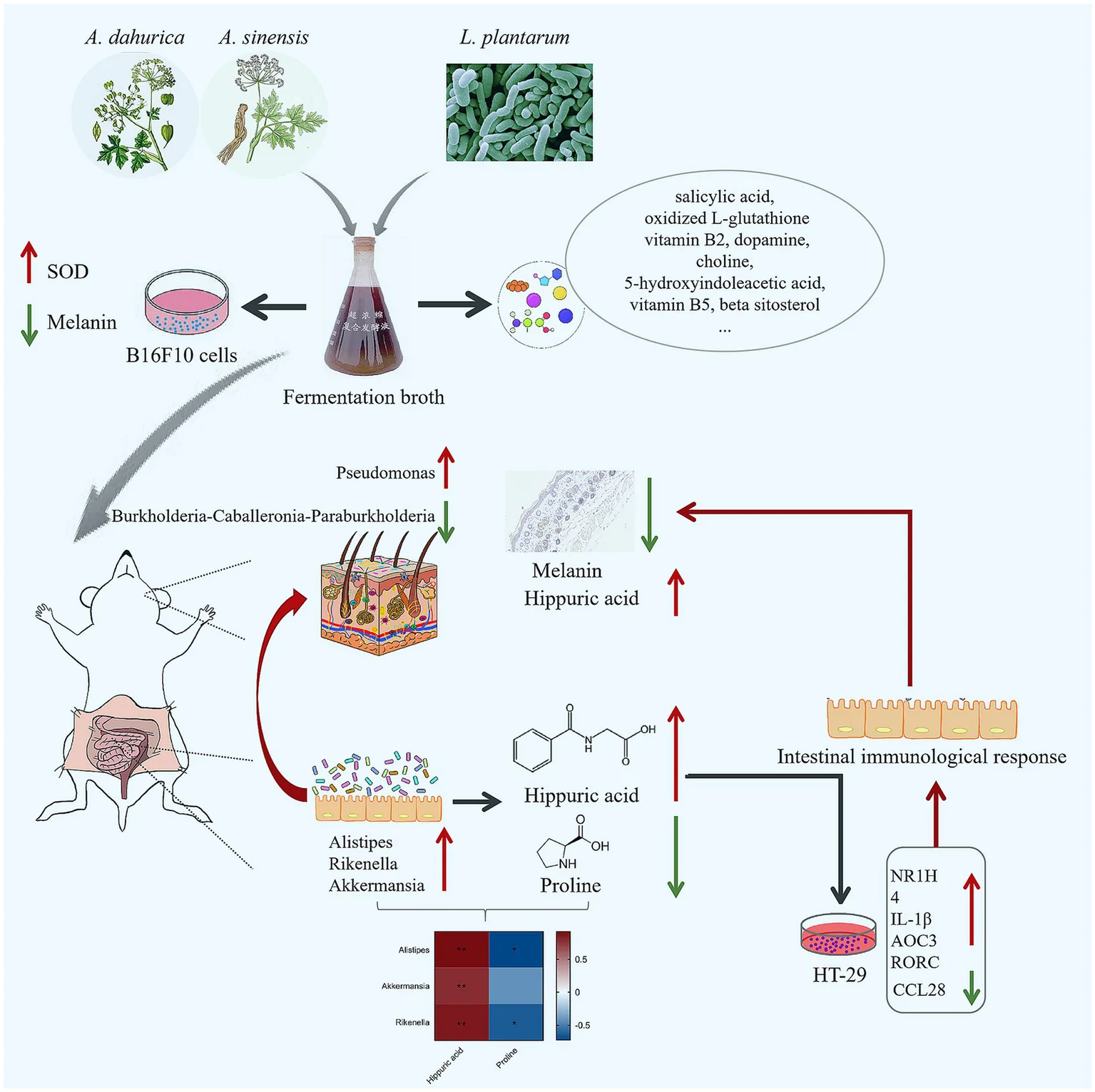

## 1 Introduction

Skin hyperpigmentation disorders, such as melasma, represent a significant cosmetic and psychological burden worldwide. Their complex etiology involves ultraviolet (UV) exposure, hormonal fluctuations, oxidative stress (1). Emerging evidence implicates dysbiosis of the gut microbiota as a novel systemic contributor to skin pathologies, framing the conceptual gut-skin axis (2, 3). This axis posits that gut-derived microbial metabolites can modulate systemic inflammation and immune responses, thereby influencing distal organs like the skin (4). Notably, distinct gut microbial dysbiosis has been observed in melasma patients (5), suggesting that modulating gut homeostasis could be a promising therapeutic avenue. Current first-line topical treatments, like hydroquinone, are often limited by side effects and recurrence (6), underscoring the urgent need for safer, systemic strategies that target the underlying pathophysiology.

Traditional Chinese Medicine (TCM) offers a rich repository of botanical resources for skin health. *Angelica dahurica* (Bai-zhi) and *A. sinensis* (Dang-gui) are particularly esteemed for their abilities to “brighten the complexion” and to “promote circulation,” respectively (7, 8). Their combination is rationally supported by both TCM theory and modern pharmacology. In TCM, *A. sinensis* activates blood circulation to resolve stasis (the core driver of melasma), whereas aromatic *A. dahurica* directs bioactive components to facial skin to clear pigment, a classic matching pair documented in ancient beauty formulas (9). Network pharmacology and *in vitro* assays confirm they exert complementary anti-melanogenic effects, with combined extracts delivering superior whitening and antioxidant capacity compared with single herb treatment (8). Modern in vitro studies have begun to validate their anti-melanogenic potential, demonstrating inhibition of tyrosinase activity and melanin synthesis (10, 11). However, the evidence remains largely phenomenological and confined to cellular models. A critical gap persists in understanding the *in vivo* mechanisms, especially how these herbs, when administered orally, exert their systemic effects on skin pigmentation. This disconnect between traditional use and a comprehensive mechanistic explanation hinders their modernization and evidence-based application.

Concurrently, probiotic fermentation has emerged as a powerful bioprocessing strategy to enhance the bioactivity and bioavailability of plant-based extracts (12). Through microbial biotransformation, complex phytochemicals are converted into more absorbable and active metabolites (e.g., phenolic acids) (13, 14), as demonstrated for other fermented botanicals (15–17). This process aligns with the concept of developing “functional foods” from traditional resources. We hypothesize that probiotic fermentation can unlock the systemic, gut-mediated therapeutic potential of *A. dahurica* and *A. sinensis*. Specifically, we propose a dual-action mechanism: the fermented extract may not only directly inhibit melanogenesis via enriched bioactive compounds but, more importantly, may systemically attenuate hyperpigmentation by remodeling the gut microbiota and elevating protective microbial metabolites, such as hippuric acid (HA), a metabolite with known anti-inflammatory properties (18, 19).

To verify this hypothesis, four sets of experiments were performed. First, we characterized metabolites in *L. plantarum*-fermented *Angelica* broth. Second, we assessed its antioxidant and anti-melanogenic activity in B16F10 cells and a UVB-induced pigmentation mouse model. Third, we analyzed shifts in gut microbiota and host metabolites upon FB intervention. Finally, we clarified how the gut microbial metabolite HA improves intestinal barrier function. By integrating TCM heritage with contemporary microbiome and metabolomic science, this work seeks to elucidate a novel, diet-based mechanism mediated by the gut-skin axis, providing a scientific rationale for the “beauty-from-within” action of these fermented traditional herbs as functional foods.

## 2 Materials and methods

### 2.1 Probiotic fermentation of the aqueous extract from *Angelica dahurica* and *Angelica sinensis*

**Figure 1:**
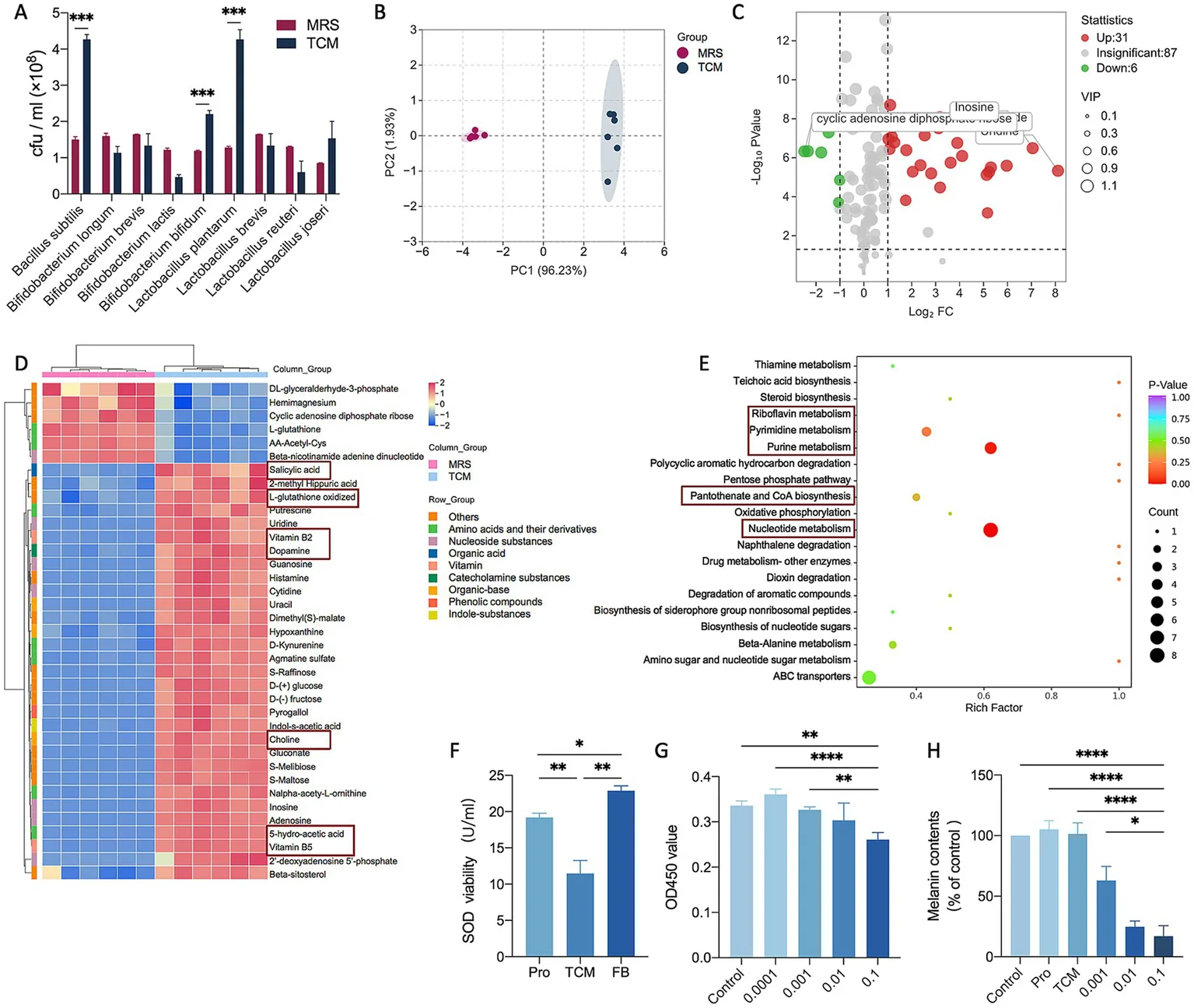
Metabolic profiling and in vitro bioactivity of probiotic-fermented *A. dahurica* and *A. sinensis*. **(A)** Bacteria growth curves in TCM-modified MRS medium. **(B)** Principal component analysis (PCA) of metabolic profiles from *L. plantarum* cultured in standard MRS or TCM medium. **(C)** Volcano plot of differentially abundant metabolites. Green, red, and gray dots represent downregulated, upregulated, and non-significant metabolites, respectively (*p* < 0.05; FC > 2 and VIP > 1). **(D)** Heatmap of hierarchical clustering for the 37 differentially abundant metabolites. **(E)** Top 20 enriched KEGG pathways for the identified metabolites. **(F)** Superoxide dismutase (SOD) activity of the probiotic suspension (Pro), unfermented TCM extract, and fermentation broth (FB). **(G)** Viability of B16F10 cells treated with serially diluted FB. **(H)** Inhibition of melanin production in B16F10 cells by FB. Data are mean ± SEM. * *p* < 0.05, ** *p* < 0.01, and **** *p* < 0.0001.

The powders of *A. dahurica* and *A. sinensis* were obtained from Huazhitang Biotechnology Co., Ltd. (Haozhou, China) and Sichuan Fuxitang Pharmaceutical Co., Ltd. (Chengdu, China), respectively. The fermentation process for *A. dahurica* and *A. sinensis* was conducted according to the methods described in our earlier research (15). In brief, a water extract was created by soaking 22.5 g of *A. dahurica* powder and 22.5 g of *A. sinensis* powder in 1 liter of water at 80 °C for 3 h. Following concentration and mixing, the extract was filtered through a sterile filter. Then, 150 mL of the filtered extract was incorporated into MRS medium powder and sterilized by autoclaving at 121 °C for 15 min. Anaerobic fermentation was performed with a 1% (v/v) inoculum of *Bacillus subtilis* (DMU22101), *Bifidobacterium longum* (DMU22158), *B. brevis* (DMU22144), *B. lactis* (DMU 22172), *B. bifidum* (DMU22167), *L. plantarum* (DMU22157), *L. brevis* (DMU22147), *L. reuteri* (DMU22115), and *L. joseri* (DMU22170) at 37 °C for 48 h. These strains are preserved in the Culture Collection of Dalian Medical University (Dalian, China). The growth of the bacterial culture was monitored by measuring the optical density at 620 nm (OD620). Among the tested strains, *L. plantarum* (DMU22157) demonstrated optimal growth in the herbal medium (as shown in Figure 1A) and was therefore selected for the production of the fermentation broth (FB) used in all subsequent experiments.

### 2.2 Detection of the differential metabolites in probiotic-fermented *Angelica dahurica* and *Angelica sinensis*

The fermentation broth was subjected to centrifugation at 12,000 g and 4 °C for 10 min, after which the supernatant was evaporated using a vacuum concentrator. The resulting extracts were then re-dissolved in an 80% methanol solution with 2-chlorobenzylamine, filtered through a 0.22 μm nylon membrane, and analyzed via UPLC–MS/MS. The chromatographic parameters were set as follows: a Waters HSS T3 column was used for separation; the mobile phase was composed of acetonitrile containing 0.1% formic acid (A) and water containing 0.1% formic acid (B). The gradient elution was programmed as follows: (0–2 min, 2% A; 2–10 min, 2% A; 10–12 min, 35% A; 12–14 min, 80% A; 14 min, 98% A; 14.1–17 min, 2% A). The flow rate was kept constant at 0.3 mL/min, the column temperature was stabilized at 40 °C, and the injection volume was set at 2 μL.

### 2.3 Evaluation of the antioxidant effect of fermentation broth

The superoxide dismutase (SOD) activity of separate probiotic suspensions (Pro), the liquid culture medium of *A. dahurica-A. sinensis* prior to fermentation (TCM), and the fermentation broth (FB) was evaluated using commercial kits (supplied by Nanjing Jiancheng Bioengineering Institute, Nanjing, China) in accordance with the manufacturer’s guidelines.

### 2.4 Cell viability assay

Murine melanoma B16-F10 cells were sourced from ATCC (Manassas, VA, U. S. A.) and were cultured in Dulbecco’s modified Eagle’s medium (DMEM) supplemented with 10% fetal bovine serum (FBS), 100 μg/mL streptomycin and 100 U/mL penicillin. The cells were kept in a humidified environment with 5% CO_2_ at 37 °C. Cell viability was assessed using the cell counting kit-8 (CCK-8) assay. B16F10 cells in the logarithmic growth phase were seeded into 96-well plates at a density of 1 × 10^5^ cells per well for 12 h. A volume of 100 μL of fermentation broth was combined with 900 μL of complete culture medium and the serially diluted by factors of 10, 10^2^, 10^3^, and 10^4^. The control group consisted of complete culture medium without any fermentation broth. After treatment for 48 h, 10 μL of CCK-8 reagent was added to each well. Following a 2-h incubation in the dark, the absorbance at 450 nm was measured using a microplate reader.

### 2.5 Melanin content assay

To assess the melanin levels in the B16F10 cells, Hosoi’s method was utilized with slight adjustments (20). B16F10 melanoma cells (1 × 10^5^ cells/well) were treated with 2 μL of various probiotic suspensions (Pro), the *A. dahurica-A. sinensis* liquid culture medium before fermentation (TCM), and the fermentation broth (FB) at different dilutions (10-fold, 10^2^-fold, 10^3^-fold) for 48 h. Following the treatment, the cells were rinsed with PBS. Then, 1 mL of sodium hydroxide solution (1 N) containing 10% DMSO was added to each well and heated at 80 °C for 1 h to dissolve the melanin. Afterward, 100 μL of the resulting lysate was transferred to a well in a 96-well plate, and the absorbance was measured at 405 nm using an ELISA plate reader. The reduction in melanin pigment production was reported as a percentage relative to the control conditions.

### 2.6 Animal experimental design

The animal study received approval from the Animal Care and Use Committee of Dalian Medical University (No. SY2023009, Approval date: 20230614). A total of thirty specific-pathogen-free (SPF) female BALB/c mice, aged 8 weeks, were sourced from the Experimental Animal House of Dalian Medical University. Following a 7-day acclimatization period, the mice were randomly divided into six groups (n = 5 per group): (1) Control (normal), (2) Melasma (model), (3) Kojic Acid (positive control, topical), (4) EI (FB, topical), (5) GI (FB, oral gavage), and (6) EI + GI (FB, combined topical and oral). Melasma was induced over a 30-day period by subjecting mice to daily UVB irradiation (*λ* = 313 nm, 1 h/day) on a shaved dorsal area, coupled with intramuscular injections of progesterone (20 mg/kg) every 2 days. Following the 30-day induction period, a 14-day treatment phase was initiated. The Kojic Acid and EI groups received daily topical application of their respective solutions (1 mL) to the dorsal skin. The GI group received FB (200 μL) via daily oral gavage. The EI + GI group received both treatments daily. Control and Melasma groups received equivalent volumes of physiological saline.

### 2.7 Assessment of skin status and histopathological sections

Twenty-four hours after the final treatment, the skin conditions on the dorsal surfaces of the mice were photographed and meticulously evaluated based on the criteria outlined in Supplementary Table S1. Subsequently, the mice were anesthetized with pentobarbital sodium and weighed using an electronic scale. Dorsal skin samples were then collected from each mouse, fixed with polyformaldehyde fixative for hematoxylin–eosin (HE) staining and immunohistochemistry (IHC) staining, and preserved as frozen skin tissue homogenate. HE staining was used to measure epidermal thickness with ImageJ software. Human melanoma black 45 (HMB45) IHC was conducted to visualize melanin granules in the epidermis and to assess the distribution and secretory activity of melanocytes. The average optical density (IOD) of the IHC staining in each group of mouse skin was quantified using Image-Pro Plus 6.0 software.

### 2.8 Measurement of NO

The levels of endothelial relaxing factor, particularly nitric oxide (NO), in the skin were determined using commercial kits (from Nanjing Jiancheng Bioengineering Institute, Nanjing, China) in accordance with the provided guidelines.

### 2.9 16S rRNA sequencing analysis

Genomic DNA was meticulously extracted from skin and fecal samples obtained from experimental mice. To amplify the V3-V4 hypervariable region of the 16S rRNA gene, polymerase chain reaction (PCR) was performed using the primer pair 341F (CCTAYGGGRBGCASCAG) and 806R (GGACTACNVGGGTWTCTAAT). The amplified products were then sequenced on the Illumina NovaSeq platform (operated by Novogene Bioinformatics Technology Co. Ltd., Beijing, China). Operational taxonomic units (OTUs) were clustered at a 97% similarity threshold using the UPARSE software. Alpha diversity indices were calculated using the Mothur software to assess species richness and evenness. *β* diversity analysis was performed using QIIME 2 to evaluate the differences in overall microbial community structure, and the results were visualized through principal coordinates analysis (PCoA) and unweighted UniFrac distance-based redundancy analysis. Additionally, linear discriminant analysis (LDA) was employed to determine the effect size of differentially abundant features, with those having an LDA ≥ 4 highlighted in the figures.

### 2.10 Analysis of fecal metabolites

Fecal samples from the Control, Melasma, and GI groups of mice were gathered. A 50 mg portion was measured and put into an Eppendorf tube, followed by the addition of 1,000 μL of an extract solution composed of methanol, acetonitrile, water in a ratio of 2: 2: 1. After preparing the sample, UPLC–MS was employed to identify and analyze the components.

### 2.11 Measurement of HA levels in serum and skin

The concentration of hippuric acid in mouse skin and serum was determined using a Mouse HA ELISA Kit (SAIPEISEN BIOLOGY, Shanghai, China) following the manufacturer’s instructions. Briefly, skin tissue was homogenized in physiological saline and centrifuged at 3000 rpm for 10 min to obtain the supernatant, while serum was collected by allowing blood to clot for 30 min followed by centrifugation at 3000 rpm for 10 min. Samples were diluted fivefold with sample diluent and assayed in duplicate. The samples and standards were incubated with HRP-conjugated detection antibody at 37 °C for 60 min, followed by washing, color development with TMB substrate for 15 min, and reaction termination. The optical density (OD) was measured at 450 nm, and the HA concentration was calculated based on the standard curve with a sensitivity of 1.0 μmol/L.

### 2.12 *In vitro* HT-29 cell cultures

This study investigated how hippuric acid influence the regulation of intestinal barrier function. HT-29 cells were cultured in high-glucose Dulbecco’s modified Eagle’s medium (Gibco, United States) supplemented with 10% heat-inactivated fetal bovine serum, 100 units/mL of penicillin, and 100 μg/mL of streptomycin. The cells were maintained at 37 °C in a humidified incubator with 5% CO_2_. During the initial phase of cell attachment period, the cells were treated with either MRS broth or hippuric acid (HA, 200 μg/mL) at 37 °C, with MRS serving as the control. After 24 h of treatment, the cells were collected for RNA extraction and subsequent RT-qPCR analysis.

### 2.13 RNA sequencing-based transcriptomics analysis

HT-29 cells treated with MRS or hippuric acid (HA, 200 μg/mL) were collected, and total RNA was extracted in accordance with the instructions provided in the TRlzol Reagent manual (Life Technologies, California, United States). Illumina RNA-seq analysis was then performed at Biomarker Technologies Co., Ltd. (*n* = 3, Beijing, China). After creating the clusters, we sequenced the library preparations on an Illumina NovaSeq platform, generating 150 bp paired-end reads. DESeq2 was utilized to conduct differential expression analysis, employing the Benjamini-Hochberg method to adjust *p*-values and control the false discovery rate. Genes identified by DESeq2 with an adjusted p-value of less than 0.01 and a fold change of at least 2 were classified as differentially expressed. Subsequently, these differentially expressed genes were subjected to pathway enrichment analysis using the clusterProfiler software in conjunction with the Kyoto Encyclopedia of Genes and Genomes (KEGG).

### 2.14 Real-time quantitative PCR

RT-PCR was conducted using 384-well plates on the QuantStudio 6 Flex real-time PCR system (Life Technologies). The reactions were prepared with ChamQ Universal SYBR qPCR Master Mix (Vazyme). The primer sequences utilized for RT-PCR are listed in Supplementary Table S2. GAPDH was employed as the internal reference gene for normalization.

### 2.15 Statistical analysis

Data are presented as the mean ± standard error of the mean (SEM). Differences between two groups were assessed using an unpaired, two-tailed Student’s t-test. For comparisons among multiple groups, one-way analysis of variance (ANOVA) was performed, followed by the Kruskal-Wallis rank sum test for post-hoc analysis. Correlation analysis was conducted using Spearman’s method. A *p*-value < 0.05 was considered statistically significant.

## 3 Results

### 3.1 Probiotic fermentation enriches the bioactivity of *Angelica* spp.

To develop a probiotic fermentation system for *A. dahurica* and *A. sinensis*, we first evaluated the growth of eight probiotic strains from the genera *Bifidobacterium* and *Lactobacillus* in a TCM-modified MRS medium. Among the tested strains, *L. plantarum* (DMU22157) exhibited optimal growth, comparable to the positive control *B. subtilis* (DMU22101) and significantly superior to its growth in standard MRS broth (Figure 1A), indicating its high efficiency in utilizing bioactive compounds from the herbal extract.

We next employed UPLC-MS/MS-based untargeted metabolomics to analyze the impact of *Angelica* extract on the intracellular metabolome of *L. plantarum.* Principal component analysis (PCA) revealed clear separation between the metabolic profiles of *L. plantarum* cultivated in the presence of absence of the TCM extract (Figure 1B). Volcano plot analysis identified 37 differentially expressed metabolites (DEMs), of which 31 were upregulated and 6 were downregulated in the TCM-supplemented group (Figure 1C). Hierarchical clustering analysis further illustrated distinct metabolite patterns between the two groups (Figure 1D). Notably, fermentation in the presence of the TCM extract significantly increased the levels of several bioactive compounds, including salicylic acid, oxidized glutathione, vitamin B2, dopamine, choline, 5-hydroxyindoleacetic acid, vitamin B5, and *β*-sitosterol. KEGG pathway enrichment analysis of the DEMs indicated significant involvement in pyrimidine metabolism, purine metabolism, nucleotide metabolism, riboflavin metabolism, and pantothenate and CoA biosynthesis (Figure 1E). This metabolic reshaping, particularly the enrichment of compounds like salicylic acid and vitamin B2 with known skin-lightening properties, provided a chemical basis for the enhanced bioactivity observed in subsequent experiments.

### 3.2 The fermentation broth exhibits potent antioxidant and anti-melanogenic activities *in vitro*

The antioxidant capacity of the FB was first evaluated by measuring its SOD-like activity. The FB demonstrated significantly higher SOD activity compared to both the *L. plantarum* suspension (Pro) and the unfermented TCM extract (Figure 1F). We next assessed the direct effects on melanogenesis using B16F10 murine melanoma cells. The CCK-8 assay revealed that the FB inhibited B16F10 cell proliferation in a dose-dependent manner (Figure 1G). Consistent with this, melanin content analysis showed that the FB significantly reduced melanin production in a dose-dependent fashion (Figure 1H), confirming its potent anti-melanogenic activity.

### 3.3 Probiotic-fermented *Angelica* spp. alleviates UVB-induced skin hyperpigmentation *in vivo*

**Figure 2:**
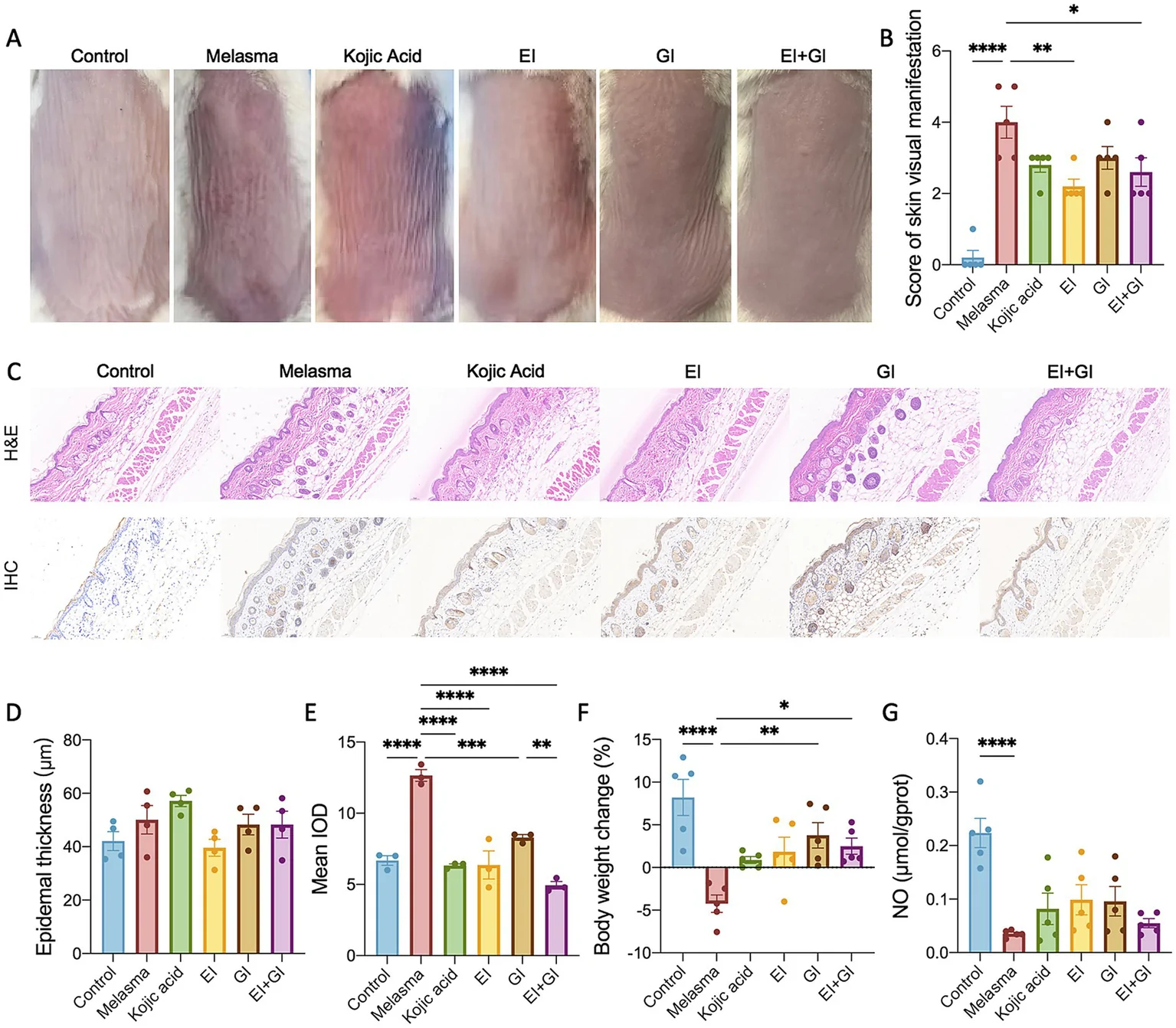
*In vivo* efficacy of probiotic-fermented *Angelica* spp. in a UVB-induced hyperpigmentation model. **(A)** Representative photographs of dorsal skin. **(B)** Clinical severity scores of skin lesions. **(C)** Representative images of H&E and HMB-45 immunohistochemical staining of skin sections. **(D)** Epidermal thickness quantification. **(E)** Mean optical density of HMB-45 staining. **(F)** Changes in body weight throughout the experiment. **(G)** Serum nitric oxide (NO) levels. Data are mean ± SEM (*n* = 5). * *p* < 0.05, ** *p* < 0.01, *** *p* < 0.001, and **** *p* < 0.0001.

We further evaluated the efficacy of probiotic-fermented *Angelica* spp. in a UVB/progesterone-induced mouse model of hyperpigmentation. Compared to the control group, model mice exhibited severe dorsal skin damage, including erythema, dryness, scabbing, and significant hyperpigmentation (Figure 2A). Both oral (GI) and topical (EI) administration of the fermentation broth, as well as their combination (EI + GI), markedly improved these skin conditions, resulting in a smoother surface with reduced pigmentation, comparable to the positive control (kojic acid) group (Figure 2A). The clinical severity scores were significantly lower in the EI and EI + GI groups than in the model group (*p* = 0.0049 and *p* = 0.0409, respectively; Figure 2B).

Histopathological analysis (H&E staining) confirmed that the model group developed epidermal hyperplasia and substantial inflammatory cell infiltration in the superficial dermis, which were alleviated by FB treatment (Figure 2C). Although epidermal thickness did not differ significantly among groups (Figure 2D), immunohistochemical staining for HMB-45 revealed a significantly higher melanocyte activity in the model group, which was effectively suppressed by FB treatment; no melanocyte ablation or focal depigmentation was observed in all treatment groups (Figure 2E). Pairwise statistical comparisons of skin lesion severity score and HMB45 staining optical density were further conducted among Melasma, EI, GI and EI + GI groups (Figures 2B,E). Relative to the Melasma group, both single EI and GI treatment significantly alleviated UVB/progesterone-induced hyperpigmentation. Although the EI + GI group exhibited numerically lower pigmentation indicators than EI or GI alone, no statistically significant differences were detected between the combined group and either monotherapy group across all measured cutaneous parameters. Furthermore, FB administration significantly mitigated the weight loss associated with the modeling process (Figure 2F). Serum NO showed an unremarkable upward trend in FB-treated mice without statistical significance (Figure 2G). Taken together, oral and topical FB both relieved UVB/progesterone-induced skin pigment lesions in the mouse model.

### 3.4 The anti-pigmentation effect is associated with gut microbiota remodeling and enrichment of beneficial taxa

**Figure 3:**
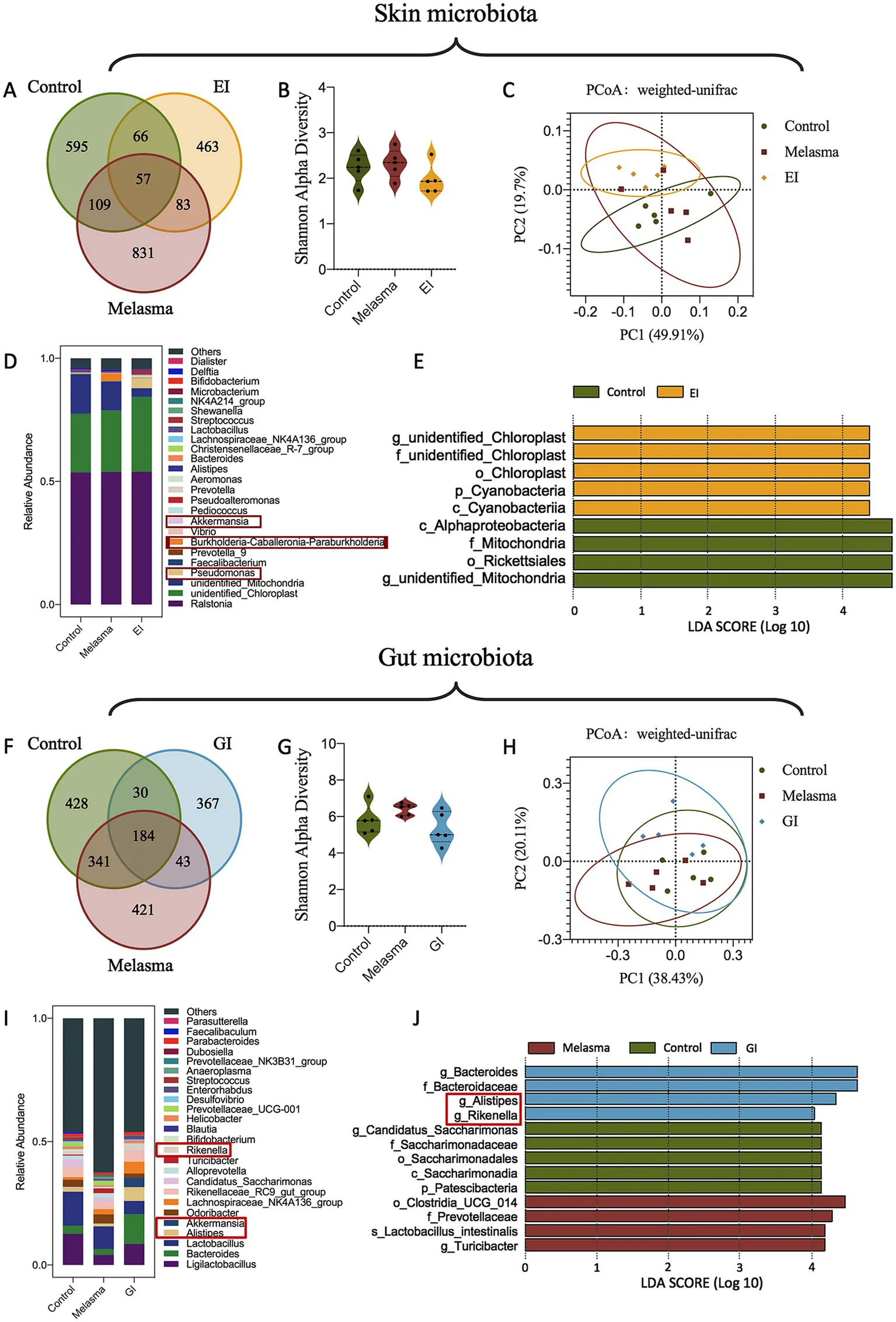
Modulation of skin and gut microbiota by probiotic-fermented *Angelica* spp. **(A,F)** Venn diagrams showing unique and shared operational taxonomic units (OTUs) among groups. **(B,G)** Alpha diversity (Shannon index). **(C,H)** Beta diversity visualized by principal coordinates analysis (PCoA) based on weighted UniFrac distances. **(D,I)** Relative abundance of bacterial communities at the genus level. **(E,J)** Taxonomic biomarkers identified by linear discriminant analysis effect size (LEfSe) (LDA score ≥ 4). Data are mean ± SEM (*n* = 5).

To determine whether the anti-pigmentation effect of fermented *Angelica* spp. involved modulation of the skin microbiota, we analyzed dorsal skin samples from Control, Melasma, and EI groups. High-throughput sequencing revealed 57 shared species among the groups, with 595, 831, and 463 unique species unique to the Control, Melasma, and EI groups, respectively (Figure 3A). No significant differences in alpha diversity (Shannon index) were observed (Figure 3B), whereas beta diversity analysis (weighted UniFrac) showed clear separation between the groups (Figure 3C). At the genus level, EI treatment increased the relative abundance of *Pseudomonas* and *Akkermansia*, while reducing that of *Burkholderia-Caballeronia-Paraburkholderia* (Figure 3D; Supplementary Figure S1). Species-level analysis indicated a significant decrease in *Lactobacillus intestinalis* in the EI group compared to the Melasma group (Supplementary Figure S1). LEfSe analysis identified *unidentified_Chloroplast* as the most discriminative feature in the EI group (Figure 3E), suggesting that topical application of the fermentation broth can ameliorate melasma-associated dysbiosis of the skin microbiota.

We next examined the effect of oral administration of the FB (GI group) on gut microbiota. Analysis of fecal samples showed 184 shared species, with 428, 421, and 367 species unique to the Control, Melasma, and GI groups, respectively (Figure 3F). Alpha diversity remained comparable across groups (Figure 3G), but beta diversity revealed distinct clustering (Figure 3H). GI treatment significantly increased the abundance of the genera *Alistipes*, *Rikenella*, and *Akkermansia* (Figure 3I; Supplementary Figure S1). At the species level, *Mucispirillum schaedleri* enriched, while *Lactobacillus intestinalis* and *Burkholderiales bacterium* YL45 were significantly reduced (Supplementary Figure S1). LEfSe analysis confirmed *Alistipes* and *Rikenella* as the most dominant genera in the GI group (Figure 3J). These results demonstrate that oral supplementation with fermented *Angelica* spp. remodels the gut microbiota by enriching beneficial taxa, supporting its role in restoring microbial homeostasis in melasma.

### 3.5 Hippuric acid is identified as a key gut microbiota-derived metabolite

**Figure 4:**
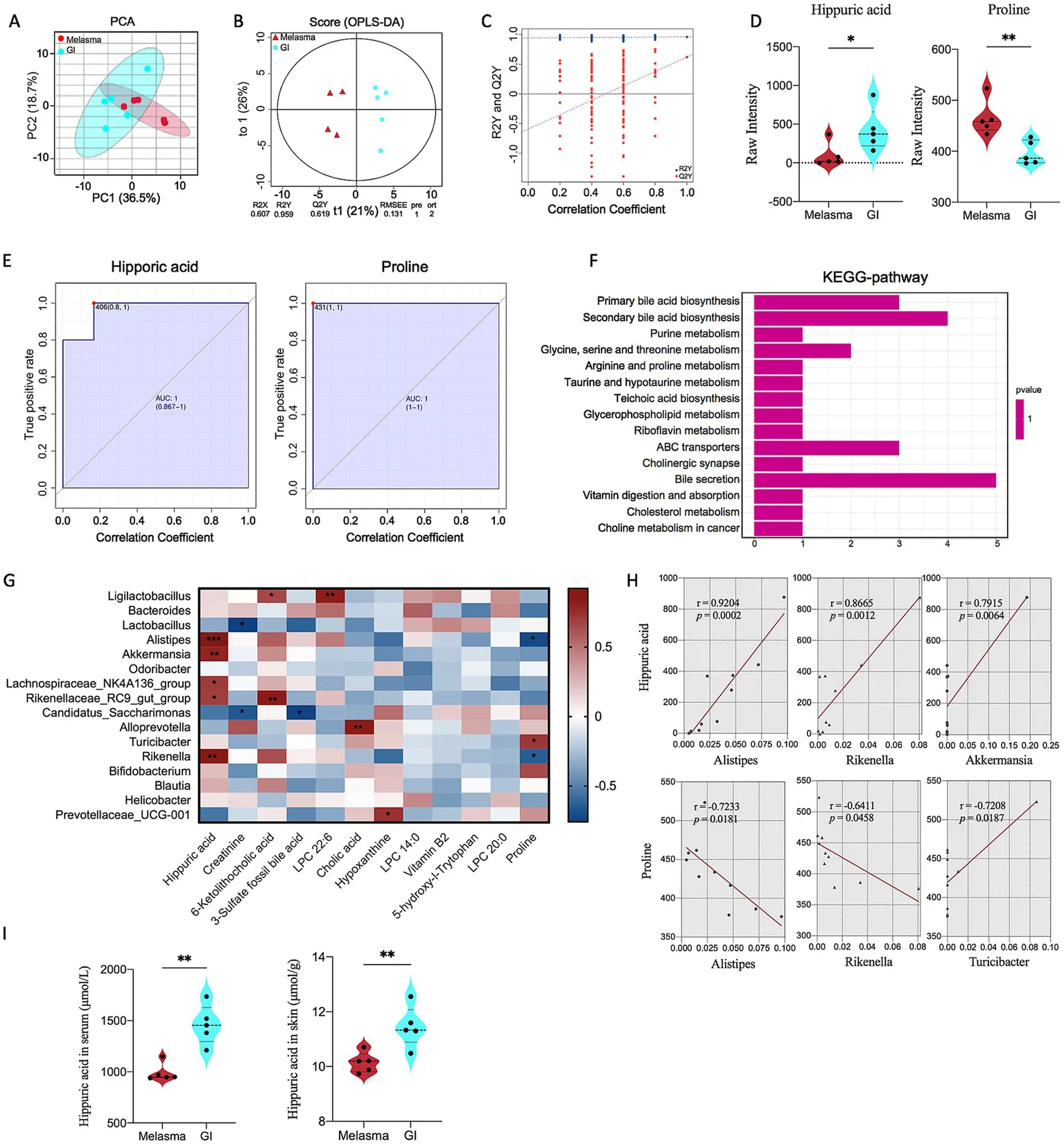
Fecal metabolomic analysis and identification of hippuric acid (HA) as a key metabolite. **(A)** PCA score plot of metabolite profiles. **(B)** OPLS-DA score plot. **(C)** Permutation test chart of the OPLS-DA model. **(D)** Relative abundance of the significantly altered metabolites hippuric acid (HA) and proline. **(E)** Receiver operating characteristic (ROC) curves for HA and proline. **(F)** KEGG pathway enrichment analysis of differentially abundant metabolites. **(G)** Spearman correlation heatmap between key metabolites and gut microbiota at the genus level. **(H)** Correlation network of HA and proline with significant bacterial genera. **(I)** Concentrations of HA in serum and skin homogenates. Data are mean ± SEM (*n* = 5). * *p* < 0.05, ** *p* < 0.01, and *** *p* < 0.0001.

Having established that oral administration of the fermented *Angelica* spp. broth (GI) reshapes the gut microbiota, we next investigated its impact on the host metabolome using untargeted LC-MS-based metabolomics. Principal component analysis (PCA) revealed a clear separation between the metabolic profiles of the Melasma and GI groups (Figure 4A). This separation was further validated by orthogonal projections to latent structures-discriminant analysis (OPLS-DA), which showed a significant distinction between the two groups (Figure 4B). The model’s reliability was confirmed by a permutation test (Figure 4C).

We then identified specific metabolites contributing to this separation. Notably, the GI group exhibited significantly elevated levels of HA and reduced levels of proline compared to the Melasma group (Figure 4D). Receiver operating characteristic (ROC) curve analysis demonstrated that both increased HA and decreased proline served as excellent biomarkers for predicting the metabolic response to fermentation broth supplementation, with high sensitivity and specificity (Figure 4E). KEGG pathway enrichment analysis of the differentially abundant metabolites highlighted 15 significantly altered pathways, including primary and secondary bile acid biosynthesis, glycine, serine, and threonine metabolism, ABC transporters, and bile secretion (Figure 4F).

To functionally link the altered gut microbiota to the changing metabolome, we performed a Spearman’s correlation analysis between 16 differentially abundant bacterial species and 12 key metabolites (Figure 4G). This analysis revealed that the beneficial genera *Alistipes* and *Rikenella*, which were enriched by the FB, showed a strong positive correlation with HA and a negative correlation with proline. *Akkermansia* was also positively correlated with HA, while *Turicibacter* was positively correlated with proline (Figure 4H). Notably, HA concentrations were markedly higher in both serum and skin of GI-treated mice versus model animals (*p* = 0.001 and *p* = 0.008; Figure 4I). The systemic accumulation of this gut-derived metabolite bridges FB-induced microbial remodeling and improved pigmentation through the gut-skin axis.

### 3.6 Hippuric acid enhances intestinal immunological barrier function *in vitro*

**Figure 5:**
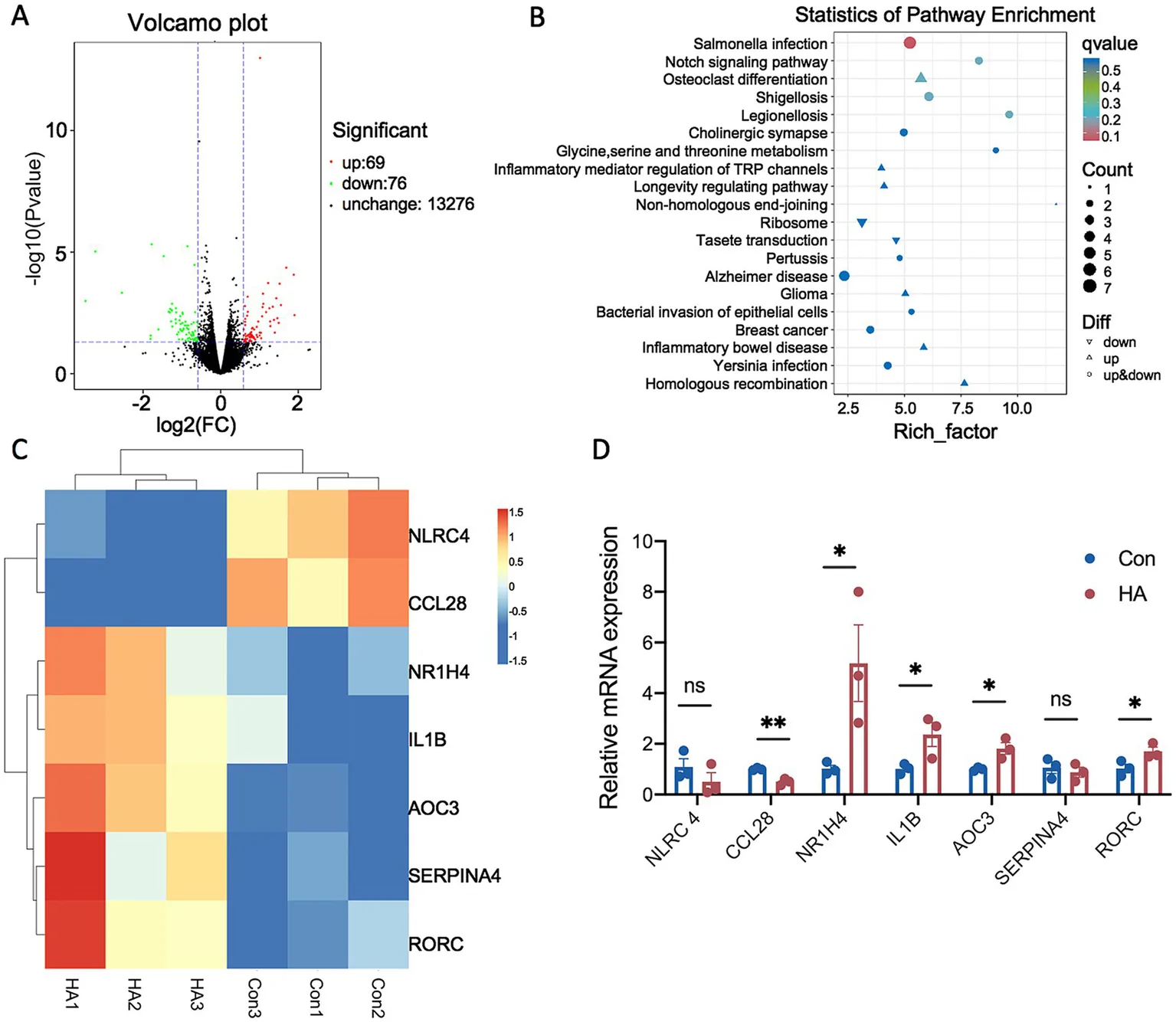
Hippuric acid (HA) enhances intestinal immunological barrier function in HT-29 cells. **(A)** Volcano plot of differentially expressed genes (DEGs) upon HA treatment (*p* < 0.05). **(B)** KEGG pathway enrichment analysis of the DEGs. **(C)** Heatmap of expression for selected DEGs related to intestinal barrier function. **(D)** Validation of key DEGs by RT-qPCR. Data are mean ± SEM (*n* = 3). * *p* < 0.05, ** *p* < 0.01, and *** *p* < 0.0001.

To explore whether HA regulates the transcriptional profile of intestinal immune barrier-related genes, we performed RNA-seq on HA-treated HT-29 IECs. Transcriptome sequencing identified a total of 23,735 genes. Comparative analysis revealed 145 differentially expressed genes (DEGs) in response to HA treatment, with 69 upregulated and 76 downregulated (Figure 5A). KEGG pathway enrichment analysis of these DEGs indicated significant involvement in pathways related to the regulation of inflammatory mediators, longevity regulation, and homologous recombination (Figure 5B). Further scrutiny identified key DEGs associated with intestinal immunological barrier function, including NR1H4, IL-1β, AOC3, SERPINA4, RORC, NLRC4, and CCL28 (Figure 5C). The expression changes of these critical genes were subsequently validated by RT-qPCR, which confirmed that HA treatment significantly upregulated NR1H4, IL-1β, AOC3, and RORC, while downregulating CCL28 (Figure 5D). Collectively, transcriptomic and RT-qPCR data indicate that HA alters the expression of a panel of core genes associated with intestinal immune homeostasis at the transcriptional level.

## 4 Discussion

**Figure 6:**
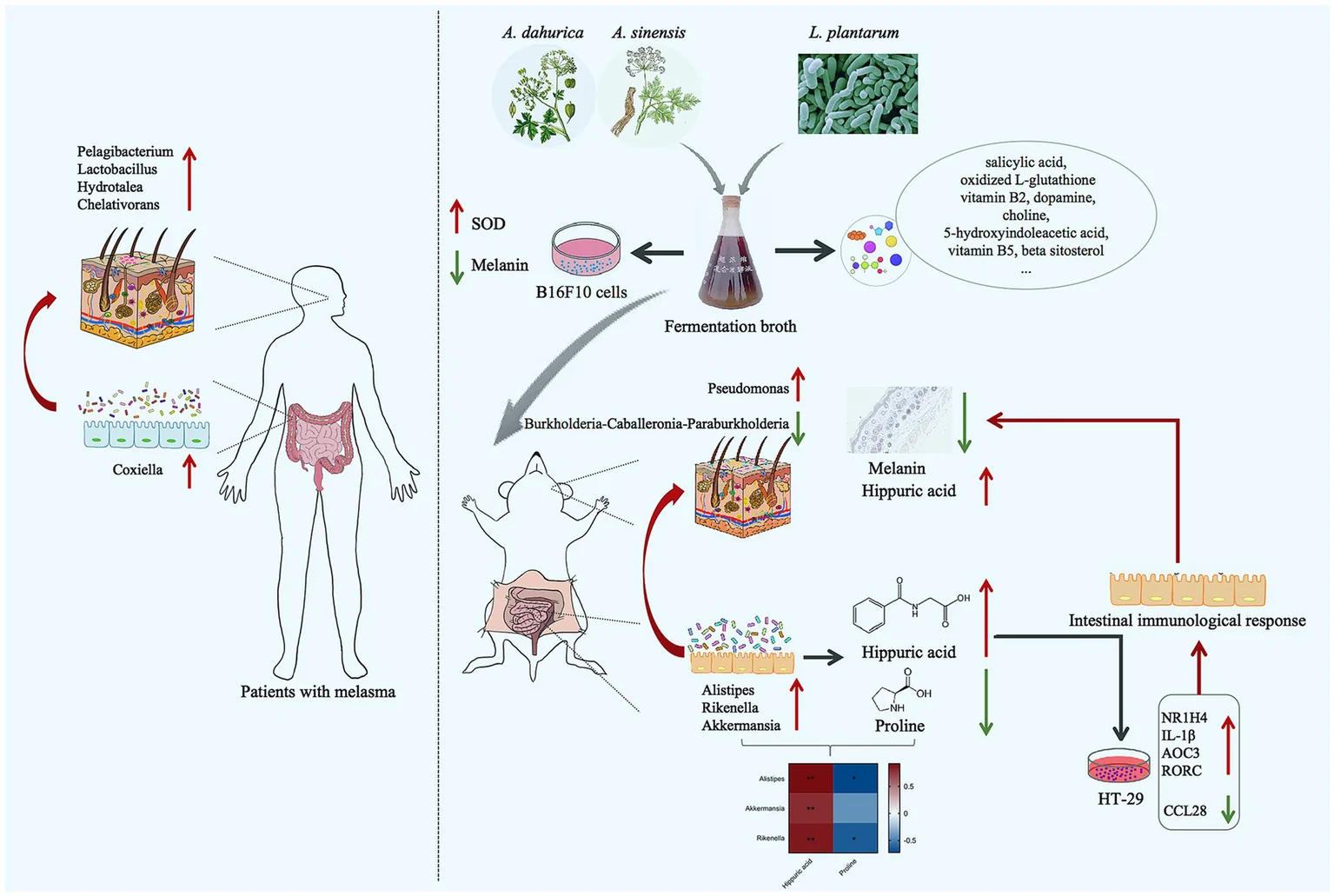
Graphical summary of the proposed dual-action mechanism. Probiotic-fermented *Angelica dahurica* and *A. sinensis* alleviate hyperpigmentation through: (1) direct inhibition of melanogenesis by enriched bioactive compounds; and (2) systemic modulation via the gut-skin axis, involving gut microbiota remodeling, elevated production of hippuric acid (HA), accompanied by transcriptional remodeling of intestinal immune-related genes, which indirectly suppresses skin melanin overproduction via reducing systemic inflammation.

Our study demonstrates that probiotic fermentation with *L. plantarum* serves as a powerful bioprocessing strategy to transform the traditional food-medicines *A. dahurica* and *A. sinensis* into a potent functional food with enhanced efficacy against hyperpigmentation. We reveal a novel dual-action mechanism: the fermented extract directly inhibits melanogenesis through enriched bioactive compounds, but, more importantly, orchestrates a systemic response by remodeling the gut microbiota and elevating the microbial metabolite HA, which remodels the transcriptional landscape of intestinal immune-related genes (Figure 6). This work provides a mechanistic elucidation of the traditional “beauty-from-within” concept and positions microbiota-targeted fermentation as a promising paradigm for modernizing herbal dermatology and functional nutrition.

Probiotic fermentation represents an effective bioprocess to unlock the bioactivity of herbal food-medicines (12, 21). Consistent with prior TCM fermentation studies (22, 23), *L. plantarum*-mediated biotransformation reshapes phytochemical profiles and produces whitening constituents including salicylic acid and riboflavin, which suppress tyrosinase and melanin synthesis (24–26). Such metabolic remodeling endows FB with *in vitro* antioxidant and anti-melanogenic activity compared with unprocessed herbs (11, 27, 28). This demonstrates that fermentation acts as a powerful bioprocessing technique to potentiate the native bioactivity of TCM.

In our UVB-progesterone pigmentation mouse model, both topical and oral FB administration relieved skin pigmentation, epidermal hyperplasia and cutaneous inflammation, yet their regulatory pathways diverged substantially. The concomitant prevention of model-associated weight loss also suggests a beneficial systemic impact. Despite marginally better pigmentation improvement shown numerically in the EI + GI combined group, pairwise statistical analysis confirmed no significant synergistic depigmentation effect relative to single oral or topical treatment. The potent standalone whitening efficacy of either GI or EI under the tested dosage and treatment cycle likely caused a ceiling effect, leaving limited room for additional therapeutic gain upon combined administration. Even without obvious synergistic effects, the dual-delivery regimen simultaneously realizes local cutaneous melanin suppression and long-distance systemic regulation via the gut-skin axis, which still supports the rationality of setting the combined intervention group in our experimental design. The therapeutic outcome was intrinsically linked to a significant restoration of microbial homeostasis at both the skin and gut levels. Topical application enriched the skin microbiota with commensals like *Pseudomonas* and *Akkermansia*, genera associated with skin health and anti-inflammatory effects (29), while reducing the pro-inflammatory-associated *Burkholderia-Caballeronia-Paraburkholderia* (30). More profoundly, oral administration induced a beneficial remodeling of the gut microbiota, marked by the enrichment of *Alistipes*, *Rikenella*, and *Akkermansia*, genera renowned for reinforcing gut barrier integrity and ameliorating inflammation (31–33).

Metabolomic analysis identified the gut microbiota-derived metabolite HA as a pivotal systemic mediator linking the oral administration of fermented *Angelica* extracts to the amelioration of skin hyperpigmentation. A significant increase in HA levels, concomitant with a decrease in proline, was observed in the GI group and served as a robust biomarker of treatment response (Figure 4). HA, a gut microbial metabolite with prominent anti-inflammatory effects (18, 19). It also aids in the elimination of intestinal uric acid (18), which encourages melanogenesis (34). The positive relation between HA and beneficial bacteria like *Alistipes*, *Rikenella*, and *Akkermansia* further emphasizes the gut microbiota’s role in influencing systemic metabolism and skin health. Polyphenol and benzoate substrates released from fermented Angelica are metabolized by gut commensals: *Alistipes* harbors complete aromatic degradation pathways to generate benzoate intermediates for HA biosynthesis (18); *Rikenella* secretes esterases to decompose complex bound polyphenols into absorbable simple phenolic fragments and boosts substrate availability (13, 14); mucin-degrading *Akkermansia* remodels intestinal mucosal microhabitats and expands the niche of polyphenol-metabolizing microbes to further raise HA production (13, 14). The coordinated metabolic capacity of these three taxa fully explains the systemic HA accumulation triggered by FB intervention. Meanwhile, decreased proline may reflect inhibited melanogenic activity, as proline biosynthesis is closely associated with melanoma progression (35). Crucially, HA levels were significantly elevated not only in the serum but also in the skin tissue of treated mice, providing direct evidence of a systemic effect mediated by this microbial metabolite. The rise in HA was positively correlated with the enrichment of beneficial genera *Alistipes*, *Rikenella*, and *Akkermansia* induced by the fermented extract, establishing a clear causal chain from gut microbiota remodeling to systemic metabolite distribution.

Notably, the dual, context-dependent biological functions of HA should be clarified to reconcile conflicting published observations. Ni et al. (36) reported that HA accumulates in epidermal melanocytes and induces ROS overproduction to trigger melanocyte damage in autoimmune vitiligo, a pathological model characterized by spontaneous melanocyte apoptosis. Distinct from vitiligo, our UVB/progesterone melasma model is dominated by excessive melanin deposition rather than melanocyte autoimmunity. In our oral FB intervention, HA is mainly produced by reshaped intestinal flora and first targets intestinal epithelium, with moderate concentrations detected in skin, which cannot reach the high epidermal HA level that causes melanotoxicity in vitiligo. Additionally, FB enriches balanced beneficial genera (*Alistipes*, *Rikenella*, *Akkermansia*) to sustain stable circulating HA levels and avoid excessive metabolite accumulation.

The functional significance of HA was further elucidated by our *in vitro* experiments. Treatment of HT-29 intestinal epithelial cells with HA differentially regulated key genes governing the intestinal immunological barrier (Figure 5). The upregulation of NR1H4 (FXR), a pivotal regulator of bile acid metabolism and inflammation (37). IL-1B, AOC3, and RORC, all critically involved in immune regulation (38–40), suggests an enhancement of host defense mechanisms. Simultaneously, the downregulation of CCL28, a chemokine implicated in the recruitment of inflammatory cells to the gut mucosa (41), points toward a suppression of pro-inflammatory signaling. These coordinated transcriptional changes strongly suggest that HA, absorbed from the gut, functions to fortify the intestinal barrier and modulate systemic immunity. From the transcriptomic profile of HA-treated HT-29 cells, we further sorted out a clear upstream-to-downstream regulatory cascade centered on NR1H4 (FXR) to interpret intestinal immune homeostasis. HA acts as an upstream agonist to activate NR1H4 in intestinal epithelial cells (18, 19). Once activated, NR1H4 recruits nuclear corepressor complexes to block NF-κB transcriptional signaling, thereby suppressing the transcription of pro-inflammatory IL-1β (37). Meanwhile, activated NR1H4 also transcriptionally inhibits CCL28, a chemokine responsible for recruiting inflammatory immune cells to intestinal mucosa (41). This hierarchical regulatory chain forms a complete homeostatic circuit: HA-induced NR1H4 activation simultaneously reduces pro-inflammatory cytokine secretion and blocks mucosal immune cell infiltration, ultimately reshaping the intestinal immune transcriptional signature to limit inflammatory signaling. The differential expression trends of NR1H4, IL-1β, and CCL28 detected in our transcriptome perfectly align with this canonical FXR-mediated gut immune regulatory axis reported in existing intestinal inflammation research, suggesting that HA-mediated transcriptional regulation of intestinal immune genes may relieve systemic inflammatory stimuli linked to UV- and hormone-driven hyperpigmentation. Consistent with published gut-skin axis research, chronic intestinal leakage and persistent systemic inflammation are well-established drivers of UV- and hormone-induced melanin overproduction (42). Beyond stabilizing intestinal immunity, circulating HA also accelerates intestinal uric acid excretion to eliminate urate, a pro-oxidant metabolite that stimulates melanocyte activity (18). After entering systemic circulation and accumulating in skin tissue, HA further relieves local cutaneous oxidative and inflammatory stress to synergistically mitigate pigmentary lesions.

Based on the above intestinal molecular mechanism driven by HA, topical and oral administration of FB exert anti-hyperpigmentation effects through fundamentally divergent pathways, which explains why only oral intervention induced a significant systemic increase in HA. For the topical EI group, FB remains confined to the epidermal surface after skin application. It directly delivers antioxidant phytochemicals to locally suppress melanogenesis and reshapes the cutaneous microbiota to relieve skin inflammation, yet the intact stratum corneum acts as a physical barrier preventing herbal components and probiotics from penetrating into the systemic circulation or reaching the intestinal tract. Without contact with gut commensal bacteria, topical FB cannot remodel gut microbial composition or stimulate intestinal microbial synthesis of HA, thus no obvious elevation of HA was observed in serum or skin tissue of EI mice. In contrast, oral GI administration delivers FB directly into the gastrointestinal niche, where co-metabolism between herbal phytochemicals and reshaped gut microbiota (including enriched *Alistipes*, *Rikenella*, and *Akkermansia*) drives robust HA production. The gut-derived HA is absorbed across the intestinal epithelium and enters systemic circulation, accumulating in peripheral skin tissue to modulate intestinal immune barrier and alleviate pigmentation via the gut-skin axis. Despite the absence of systemic HA elevation in the topical group, local regulation of skin microbiota and direct anti-melanogenic activity still conferred mild pigment-improving effects. Consistent with this divergent mechanism, the combined EI + GI group achieved the optimal therapeutic outcome, combining local cutaneous inhibition of melanin and long-distance systemic regulation mediated by gut-derived HA.

Within the context of TCM theory, which posits that skin health is a reflection of internal balance, HA emerges as a critical scientific entity. It mechanistically explains the “internal” action of the fermented herbs: by modulating the gut microbiota to raise HA levels, the intervention reshapes intestinal immune gene transcription to reduce systemic inflammatory tone, potentially reducing the systemic inflammatory tone that can drive melanogenesis. This provides a novel molecular rationale for the traditional “beauty-from-within” concept. The reduction in proline, a precursor linked to melanoma progression (35), further underscores a multi-targeted metabolic shift favoring the suppression of pigmentation. Therefore, HA is not merely a biomarker but a key functional effector that bridges the gap between gut ecology and skin physiology, offering a novel, metabolite-targeted perspective for the treatment of hyperpigmentation disorders through the gut-skin axis.

The present study elucidates a novel, dual-mechanism underlying the efficacy of probiotic-fermented *A. dahurica* and *A. sinensis* against hyperpigmentation (Figure 6). Diverging from prior investigations that focused on singular pathways, we demonstrate that the fermented extract concurrently directly inhibits melanogenesis *in vitro* and orchestrates a systemic response via the gut-skin axis *in vivo*. This systemic effect is mediated through gut microbiota remodeling and a consequent rise in the microbial metabolite HA, which we identified as a critical effector enhancing intestinal barrier immunity. The elevation of HA in the systemic circulation and skin tissue establishes it as a pivotal biomarker linking gut health to skin homeostasis, providing a mechanistic explanation for the traditional “beauty-from-within” concept. Despite these insights, certain limitations warrant acknowledgment. The precise molecular targets through which HA modulates melanogenesis remain to be fully delineated. Furthermore, the clinical translatability of these findings requires validation in human trials. Future work will focus on addressing these points to further clarify the mechanisms and enhance the clinical applicability of this intervention. In addition to the above limitations, several practical translational hurdles should be noted. Significant differences in gut microbiota composition and metabolic function between mice and humans may reduce the HA-mediated gut-skin axis effect when translated to human subjects. Besides, the mouse oral dosage cannot be directly applied to humans, and standardized human equivalent doses need to be determined for functional food development. Meanwhile, liquid fermented *Angelica* broth suffers from poor storage stability, as probiotic activity and active phytochemicals degrade easily during preservation, limiting industrial production. Future work will adopt human fecal fermentation models, carry out dosage conversion trials, and optimize stabilizing formulations such as microcapsules. Human clinical trials are also needed to verify its skin-lightening efficacy in populations with pigmentary disorders.

## 5 Conclusion

In conclusion, by integrating traditional herbal wisdom with modern probiotic biotechnology and systems biology, this study not only deciphers the scientific basis of the “beauty-from-within” concept but also establishes probiotic-fermented *Angelica* spp. as a promising, gut-microbiota-targeting dietary strategy for managing hyperpigmentation disorders. Our approach offers a translatable model for leveraging fermentation to unlock the functional potential of traditional phytochemical resources in promoting skin and systemic health.

## Data Availability

The datasets generated and analyzed during the current study are deposited in the GenBank Sequence Read Archive (SRA) with the accession number PRJNA1469499.

## Glossary

ANOVA: Analysis of Variance
B16F10: B16F10 melanoma cells
BMI: Body Mass Index
CCK-8: Cell Counting Kit-8
DMEM: Dulbecco’s Modified Eagle’s Medium
DNA: Deoxyribonucleic Acid
EI: Epidermal Intervention
FB: Fermentation Broth
GI: Gastrointestinal
HA: Hippuric Acid
HE: Hematoxylin–Eosin
HMB45: Human Melanoma Black 45
IHC: Immunohistochemistry
KEGG: Kyoto Encyclopedia of Genes and Genomes
LEfSe: Linear Discriminant Analysis Effect Size
mMASI: Modified Melasma Area and Severity Index
NO: Nitric Oxide
OTUs: Operational Taxonomic Units
PCoA: Principal Coordinates Analysis
PCA: Principal Component Analysis
ROC: Receiver Operating Characteristic
RT-qPCR: Real-Time Quantitative PCR
SOD: Superoxide Dismutase
TCM: Traditional Chinese Medicine
UPLC-MS/MS: Ultra-Performance Liquid Chromatography Tandem Mass Spectrometry
UV: Ultraviolet
V3-V4: 16S rRNA gene V3-V4 region

## References

[1] Liao T Luo R Deng Y Yang H Du Y. Targeting Melasma: innovations in pigment deposition and Photoaging in cosmetic dermatology. J Cosmet Dermatol. (2026) 25:e70665. doi: 10.1111/jocd.70665, 41508749 PMC12783984

[2] Mahmud MR Akter S Tamanna SK Mazumder L Esti IZ Banerjee S . Impact of gut microbiome on skin health: gut-skin axis observed through the lenses of therapeutics and skin diseases. Gut Microbes. (2022) 14:2096995. doi: 10.1080/19490976.2022.2096995, 35866234 PMC9311318

[3] Liscano Y Muñoz Morales D Suarez Daza F Vidal Cañas S Martinez Guevara D Artunduaga Cañas E. Microbial interventions for inflammatory skin diseases: a systematic review and meta-analysis of atopic dermatitis and psoriasis. Microorganisms. (2025) 13:2416. doi: 10.3390/microorganisms13112416, 41304102 PMC12654813

[4] Yang W Cong Y. Gut microbiota-derived metabolites in the regulation of host immune responses and immune-related inflammatory diseases. Cell Mol Immunol. (2021) 18:866–77. doi: 10.1038/s41423-021-00661-4, 33707689 PMC8115644

[5] Liu C He D Yu A Deng Y Wang L Song Z. Correlation analysis between gut microbiota characteristics and melasma. Front Microbiol. (2022) 13:1051653. doi: 10.3389/fmicb.2022.1051653, 36466650 PMC9714260

[6] AlJabr A AlAnazi AMI AlEtebi RAA. Tranexamic acid for hyperpigmentation disorders: a literature review on efficacy and safety in melasma and PIH. J Cosmet Dermatol. (2026) 25:e70692. doi: 10.1111/jocd.70692, 41601401 PMC12848551

[7] Liu X Li X He W Han X Guo S Xiao T . Integrated in vitro - in vivo evaluation of Angelica sinensis essential oil as an anti-acne agent: mechanistic insights into bioactive compounds and multi-target therapeutic actions. J Ethnopharmacol. (2026) 359:121114. doi: 10.1016/j.jep.2025.121114, 41482092

[8] Liang Y Chen J Fu C Dai X Jiang L Lei X. Network pharmacology approach to explore the skin-lightening compounds and potential mechanisms of Chinese herbal medicines. J Cosmet Dermatol. (2025) 24:e70562. doi: 10.1111/jocd.70562, 41351313 PMC12680926

[9] Ko CY Chao J Chen PY Su SY Maeda T Lin CY . Ethnobotanical survey on skin whitening prescriptions of traditional Chinese medicine in Taiwan. Front Pharmacol. (2021) 12:736370. doi: 10.3389/fphar.2021.736370, 34916932 PMC8670535

[10] Cho YH Kim JH Park SM Lee BC Pyo HB Park HD. New cosmetic agents for skin whitening from Angelica dahurica. J Cosmet Sci. (2006) 57:11–21. 16676120

[11] Lam RY Lin ZX Sviderskaya E Cheng CH. Application of a combined sulphorhodamine B and melanin assay to the evaluation of Chinese medicines and their constituent compounds for hyperpigmentation treatment. J Ethnopharmacol. (2010) 132:274–9. doi: 10.1016/j.jep.2010.08.027, 20723597

[12] Yang HY Han L Lin YQ Li T Wei Y Zhao LH . Probiotic fermentation of herbal medicine: progress, challenges, and opportunities. Am J Chin Med. (2023) 51:1105–26. doi: 10.1142/s0192415x23500519, 37357176

[13] Lu X Li J Ma Y Khan I Yang Y Li Y . Fermented Angelica sinensis activates Nrf2 signaling and modulates the gut microbiota composition and metabolism to attenuate D-gal induced liver aging. Food Funct. (2023) 14:215–30. doi: 10.1039/d2fo01637k, 36477974

[14] Wang Z Jin X Zhang X Xie X Tu Z He X. From function to metabolome: Metabolomic analysis reveals the effect of probiotic fermentation on the chemical compositions and biological activities of *Perilla frutescens* leaves. Front Nutr. (2022) 9:933193. doi: 10.3389/fnut.2022.933193, 35898707 PMC9309800

[15] Li Y Lei Z Guo Y Liu Y Guo X Wang X . Fermentation of Ganoderma lucidum and Raphani semen with a probiotic mixture attenuates cyclophosphamide-induced immunosuppression through microbiota-dependent or -independent regulation of intestinal mucosal barrier and immune responses. Phytomedicine. (2023) 121:155082. doi: 10.1016/j.phymed.2023.155082, 37722243

[16] Li Y Liu H Qi H Tang W Zhang C Liu Z . Probiotic fermentation of Ganoderma lucidum fruiting body extracts promoted its immunostimulatory activity in mice with dexamethasone-induced immunosuppression. Biomed Pharmacother. (2021) 141:111909. doi: 10.1016/j.biopha.2021.111909, 34328088

[17] Chen Y Wei X Rui B Du Y Lei Z Guo X . Probiotic fermentation of Astragalus membranaceus and Raphani semen ameliorates cyclophosphamide-induced immunosuppression through intestinal short-chain fatty acid-dependent or -independent regulation of B cell function. Biology (Basel). (2025) 14:312. doi: 10.3390/biology14030312, 40136568 PMC12077259

[18] Xu YX Liu LD Zhu JY Zhu SS Ye BQ Yang JL . *Alistipes indistinctus*-derived hippuric acid promotes intestinal urate excretion to alleviate hyperuricemia. Cell Host Microbe. (2024) 32:366–81.e9. doi: 10.1016/j.chom.2024.02.001, 38412863

[19] Yang Y Huang S Liao Y Wu X Zhang C Wang X . Hippuric acid alleviates dextran sulfate sodium-induced colitis via suppressing inflammatory activity and modulating gut microbiota. Biochem Biophys Res Commun. (2024) 710:149879. doi: 10.1016/j.bbrc.2024.149879, 38579536

[20] Hosoi J Abe E Suda T Kuroki T. Regulation of melanin synthesis of B16 mouse melanoma cells by 1 alpha, 25-dihydroxyvitamin D3 and retinoic acid. Cancer Res. (1985) 45:1474–8. 2983883

[21] Ma J Wang J Wan Y Wang S Jiang C. Probiotic-fermented traditional Chinese herbal medicine, a promising approach to maintaining the intestinal microecology. J Ethnopharmacol. (2025) 337:118815. doi: 10.1016/j.jep.2024.118815 39270882

[22] Li J Ma Y Li X Wang Y Huo Z Lin Y . Fermented Astragalus and its metabolites regulate inflammatory status and gut microbiota to repair intestinal barrier damage in dextran sulfate sodium-induced ulcerative colitis. Front Nutr. (2022) 9:1035912. doi: 10.3389/fnut.2022.1035912, 36451737 PMC9702530

[23] Guo X Yan Z Wang J Fan X Kang J Niu R . Effect of traditional chinese medicine (TCM) and its fermentation using *Lactobacillus plantarum* on ceftriaxone sodium-induced dysbacteriotic diarrhea in mice. Chin Med. (2022) 17:20. doi: 10.1186/s13020-022-00575-x, 35139871 PMC8827261

[24] Liu J Jiang R Zhou J Xu X Sun Z Li J . Salicylic acid in ginseng root alleviates skin hyperpigmentation disorders by inhibiting melanogenesis and melanosome transport. Eur J Pharmacol. (2021) 910:174458. doi: 10.1016/j.ejphar.2021.174458, 34480884

[25] Kim B Hwang JS Kim HS. N-Nicotinoyl dopamine inhibits skin pigmentation by suppressing of melanosome transfer. Eur J Pharmacol. (2015) 769:250–6. doi: 10.1016/j.ejphar.2015.11.025, 26597116

[26] Machado D Shishido SM Queiroz KC Oliveira DN Faria AL Catharino RR . Irradiated riboflavin diminishes the aggressiveness of melanoma in vitro and in vivo. PLoS One. (2013) 8:e54269. doi: 10.1371/journal.pone.0054269, 23342114 PMC3546980

[27] Wang GH Chen CY Tsai TH Chen CK Cheng CY Huang YH . Evaluation of tyrosinase inhibitory and antioxidant activities of Angelica dahurica root extracts for four different probiotic bacteria fermentations. J Biosci Bioeng. (2017) 123:679–84. doi: 10.1016/j.jbiosc.2017.01.003, 28254340

[28] Yang H Li Q. Optimization of extraction process and the antioxidant activity spectrum-effect relationship of Angelica dahurica. Biomed Chromatogr. (2022) 36:e5322. doi: 10.1002/bmc.5322, 34989001

[29] Cogen AL Nizet V Gallo RL. Skin microbiota: a source of disease or defence? Br J Dermatol. (2008) 158:442–55. doi: 10.1111/j.1365-2133.2008.08437.x, 18275522 PMC2746716

[30] Zou D Lu X Song F Zhong X Chen H Zhang J . Characteristics of bacterial community in eyelashes of patients with Demodex blepharitis. Parasit Vectors. (2024) 17:64. doi: 10.1186/s13071-024-06122-x, 38355686 PMC10868039

[31] Bi Z Chen J Chang X Li D Yao Y Cai F . ADT-OH improves intestinal barrier function and remodels the gut microbiota in DSS-induced colitis. Front Med. (2023) 17:972–92. doi: 10.1007/s11684-023-0990-1, 37507636

[32] Mou D Ding D Pu J Zhou P Cao E Zhang X . Effects of dietary pretreatment with all-trans lycopene on lipopolysaccharide-induced Jejunal inflammation: a multi-pathway phenomenon. Foods. (2025) 14:794. doi: 10.3390/foods14050794, 40077496 PMC11898642

[33] Zheng M Han R Yuan Y Xing Y Zhang W Sun Z . The role of *Akkermansia muciniphila* in inflammatory bowel disease: current knowledge and perspectives. Front Immunol. (2022) 13:1089600. doi: 10.3389/fimmu.2022.1089600, 36685588 PMC9853388

[34] Kim NH Lee AY. Growth factors upregulated by uric acid affect guanine deaminase-induced Melanogenesis. Biomol Ther (Seoul). (2023) 31:89–96. doi: 10.4062/biomolther.2022.137, 36549672 PMC9810452

[35] Kardos GR Wastyk HC Robertson GP. Disruption of proline synthesis in melanoma inhibits protein production mediated by the GCN2 pathway. Mol Cancer Res. (2015) 13:1408–20. doi: 10.1158/1541-7786.Mcr-15-0048, 26082174 PMC5238710

[36] Ni Q Xia L Huang Y Yuan X Gu W Chen Y . Gut microbiota dysbiosis orchestrates vitiligo-related oxidative stress through the metabolite hippuric acid. Microbiome. (2025) 13:112. doi: 10.1186/s40168-025-02102-0, 40329424 PMC12054231

[37] Albrecht S Fleck AK Kirchberg I Hucke S Liebmann M Klotz L . Activation of FXR pathway does not alter glial cell function. J Neuroinflammation. (2017) 14:66. doi: 10.1186/s12974-017-0833-6, 28351411 PMC5371249

[38] Zhang L Li J Zhang Q Gao J Zhao K Asai Y . An integrative analysis of single-cell RNA-seq, transcriptome and Mendelian randomization for the identification and validation of NAD(+) metabolism-related biomarkers in ulcerative colitis. Int Immunopharmacol. (2025) 145:113765. doi: 10.1016/j.intimp.2024.113765, 39647286

[39] Özcan Ö Akyol Ö Akyol A. Amine oxidase, copper containing 3 (Aoc3) knockout mice are more prone to DSS-induced colitis and colonic tumorigenesis. In Vivo. (2024) 38:2300–9. doi: 10.21873/invivo.13695, 39187313 PMC11363779

[40] Lee JS Tato CM Joyce-Shaikh B Gulen MF Cayatte C Chen Y . Interleukin-23-independent IL-17 production regulates intestinal epithelial permeability. Immunity. (2015) 43:727–38. doi: 10.1016/j.immuni.2015.09.003, 26431948 PMC6044435

[41] Lee DS Lee KL Jeong JB Shin S Kim SH Kim JW. Expression of chemokine CCL28 in ulcerative colitis patients. Gut Liver. (2021) 15:70–6. doi: 10.5009/gnl19273, 32102131 PMC7817927

[42] Cornachini CK De Souza LP Salvador DGM Silva KD Marinho de Souza J. Gut microbiota-skin axis in melasma: microbial metabolites and hormonal crosstalk. FEMS Microbiol Lett. (2026) 373:373. doi: 10.1093/femsle/fnag036, 41921062

