# Supplementary material for: A functional food approach: probiotic-fermented Angelica spp. improves skin hyperpigmentation via gut-skin axis and its microbial metabolite hippuric acid
Source: Front Nutr. 2026 Aug 18;13:1890406. doi: 10.3389/fnut.2026.1890406 (PMC13529979; doi:10.3389/fnut.2026.1890406)
Supplement: Supplementary file 1 [file Supplementary_file_1.docx]

**Supplementary figure**

**Figure S1 Differential microbiota of skin and gut microbiota among the Control, Melasma, and EI (or GI) groups.**
