# Supplementary material for: A functional food approach: probiotic-fermented Angelica spp. improves skin hyperpigmentation via gut-skin axis and its microbial metabolite hippuric acid
Source: Front Nutr. 2026 Aug 18;13:1890406. doi: 10.3389/fnut.2026.1890406 (PMC13529979; doi:10.3389/fnut.2026.1890406)
Supplement: Supplementary file 2 [file Supplementary_file_2.docx]

**Supplementary tables**

**Table S1 Scoring criteria**

| Symptom | 0 | 1 | 2 | 3 |
| --- | --- | --- | --- | --- |
| Pigmentation | None | Light brown | Moderate brown | Dark brown |
| Squama | None | Slight | Moderate | Marked |
| Erythema | None | Mild | Moderate | Severe with edema, crusting, and necrosis |
| Folds | None | Slight | Moderate | Marked |
| Elasticity | Good | Slightly impaired | Severely impaired | Completely absent |
| Thickness | Normal | Mildly thickened | Moderately thickened | Severely thickened |

**Table S2 Primer sequences**

| Gene | Forward | Reverse |
| --- | --- | --- |
| hβ-actin | 5’- ATTGCCGACAGGATGCAGAA-3’ | 5’- AAGCATTTGCGGTGGACGAT-3’ |
| hNLRC4 | 5’- CGTCCAATGTGAGTGTGCCT-3’ | 5’- GGTCACACAGGGCAAACAGA-3’ |
| hNR1H4 | 5’- TTCTGGGGCTTTGCGGTTT-3’ | 5’- CAGCATCTCTTCCTTGGCTCT-3’ |
| hIL1B | 5’- AACAGCGAGGGAGAAACTGG-3’ | 5’- GGTCGGAGATTCGTAGCTGG-3’ |
| hAOC3 | 5’- TCCTTCCCACCCTTAGTCCC-3’ | 5’- AGCACGAGGATTGTCTTCTGG-3’ |
| hRORC | 5’-GAGAAGGACAGGGAGCCAAG-3’ | 5’-GGTTCCTGTTGCTGCTGTTG-3’ |
| hCCL28 | 5’-GGCCCTACATGCCTCAGAA-3’ | 5’-GGCTACATTTGCATACCGCA-3’ |
| hSERPINA4 | 5’-AGTGGGGTGTCTTCTTGGGA-3’ | 5’-CCCAGGAGGACTGGTCAAAC-3’ |
